# Update on the Use of Infrared Thermography in the Early Detection of Diabetic Foot Complications: A Bibliographic Review

**DOI:** 10.3390/s24010252

**Published:** 2023-12-31

**Authors:** Marina Faus Camarena, Marta Izquierdo-Renau, Iván Julian-Rochina, Manel Arrébola, Manuel Miralles

**Affiliations:** 1 Nursing Department, University of Valencia, 46010 Valencia, Spain; marinafauscama@gmail.com (M.F.C.); marta.izquierdo-renau@uv.es (M.I.-R.); 2 Frailty Research Organized Group (FROG), University of Valencia, 46010 Valencia, Spain; 3 Department Angiology and Vascular Surgery, La Fe University and Polytechnic Hospital, 46026 Valencia, Spain; manelarrebola@gmail.com (M.A.); mirallesmanher@gva.es (M.M.); 4 Department of Surgery, University of Valencia, 46010 Valencia, Spain; 5 Haemostasis, Thrombosis, Arteriosclerosis and Vascular Biology Research Group, Medical Research Institute, Hospital La Fe, 46026 Valencia, Spain

**Keywords:** infrared thermography, thermal imaging, diabetic foot ulcer, diabetic neuropathy, diabetic patients, peripherical arterial disease, foot at risk

## Abstract

Foot lesions are among the most frequent causes of morbidity and disability in the diabetic population. Thus, the exploration of preventive control measures is vital for detecting early signs and symptoms of this disease. Infrared thermography is one of the complementary diagnostic tools available that has proven to be effective in the control of diabetic foot. The last review on this topic was published in 2015 and so, we conducted a bibliographic review of the main databases (PubMed, the Web of Science, Cochrane library, and Scopus) during the third quarter of 2023. We aimed to identify the effectiveness of infrared thermography as a diagnostic element in pre-ulcerous states in diabetic patients and to detect diabetic foot ulcer complications. We obtained a total of 1199 articles, 26 of which were finally included in the present review and published after 2013. After analyzing the use of infrared thermography in diabetic patients both with and without ulcers, as well as in healthy individuals, we concluded that is an effective tool for detecting early-stage ulcers in diabetic foot patients.

## 1. Introduction

Foot lesions are among the most frequent causes of morbidity and disability in the population with diabetes and are the most common reason for hospital admission and decreased patient quality of life. Indeed, in this population, there is a 40–70% probability of requiring a lower limb amputation [1]. Exploration and preventive control of this pathology are vital to detect early signs and symptoms that, in the long term, can promote the appearance of ulcers. This exploration must be performed at least once in the absence of risk factors and once every six months if there is any risk of ulcers [2]. Therefore, it is especially important to follow a protocol for these patients, in which the anamnesis and clinical history play a substantial role.

Different complementary telemedical methods are available to aid the diagnosis of diabetic foot, of which infrared thermography is one of the most important [3]. This technique was first used for military applications at the beginning of the 20th century. However, infrared thermography soon transferred to biomedical fields and started being used for the non-invasive diagnosis of vascular disease, fever, breast cancer, and in the analysis of inflammatory arthritis, osteoarthritis, and other pathologies [4].

Infrared thermography is a safe, repeatable, contactless, and non-invasive procedure that measures and maps the temperature distribution radiating from body surfaces [5]. An infrared camera identifies and monitors the amount of radiation emitted and translates this value into a temperature. These projections allow identification of the heat radiating away from the body [3,6] and produce images with specific physiological thermal patterns that can be collected according to specific standards, thereby allowing the quality of this technique to increase in the future [6].

Feet temperature variation in neuropathic patients is a predictive element of the ulcer appearance, so infrared thermography, due to its characteristics and easy use, is a good tool to detect this temperature difference [7].

The aim of this systematic review was to define the effectiveness of infrared thermography as a diagnosis tool for pre-ulcerous states in patients with type-2 diabetic mellitus and to detect ulcer complications in patients with diabetic foot.

Based on the results obtained, we will try to provide relevant information for health professionals who use this technique, speeding up and helping in decision-making in patients who may develop an ulcer or its reappearance. However, there is some controversy in generalizing these statements, so more studies are needed to generalize the findings.

## 2. Materials and Methods

Following the preferred reporting items for systematic review and meta-analysis descripts (PRISMA) guidelines, we conducted a systematic review of the academic literature on infrared thermography as a tool for diagnosing diabetic foot. We followed the population, intervention, control, and outcomes (PICO) format (Table 1) to formulate the basis of this research. 

We consulted the Descriptors in Health Science (DeCS) and Medical Subject Headings (MeSH) terms to devise the following PubMed search strategy: ((((“Diabetic Foot”[Title/Abstract]) OR (diabetic foot[MeSH Terms])) OR ((“Diabetic Neuropat hies”[Title/Abstract]) OR (diabetic neuropathies[MeSH Terms])))) AND (((“thermal imaging”[Title/Abstract]) OR (differential thermal analysis[MeSH Terms])) OR (analyses, differential thermal[MeSH Terms])). The results were subsequently summarized in databases, as shown in Table 2. 

We searched the main health science databases including PubMed, the Web of Science (WoS), Scopus, and the Cochrane library during the third quarter of 2023. The inclusion criteria were articles about infrared thermography conducted in humans with and without diabetes and ulcers, published from 2013 to 2023 in English or Spanish. Some publications such as editorials, editor’s letters, reviews, systematic reviews, meta-analyses, books or books chapters and conference reports were excluded. Of note, no Cochrane reviews were included because none met the inclusion criteria. Once the screened publications were obtained, we read the publications and reviewed their quality by employing the PEDro scale [8].

## 3. Results

**Figure 1:**
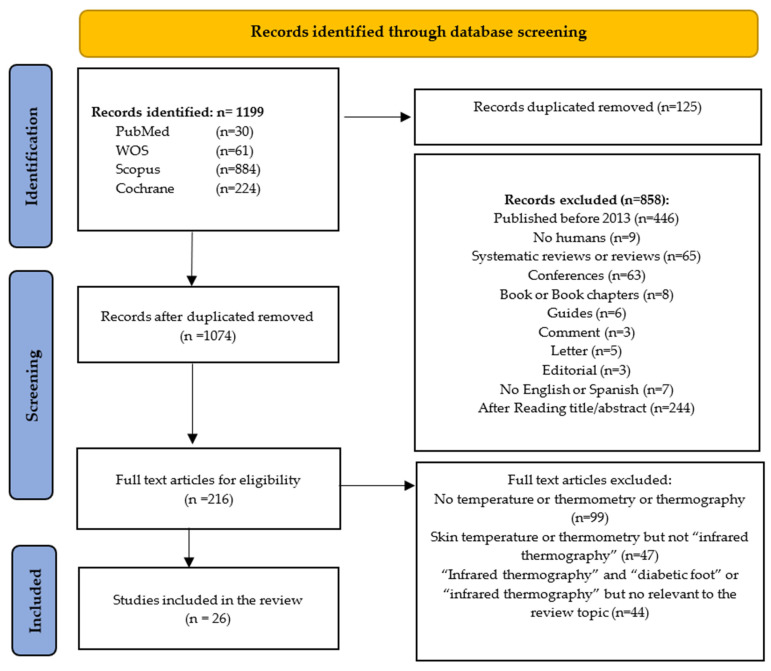
PRISMA study selection flowchart.

Our search strategy obtained a total of 1199 publications (30 in PubMed, 61 in the WoS, 224 in the Cochrane library, and 884 in Scopus). After applying the inclusion criteria, 26 valid citations were reviewed, as reflected in the PRISMA flowchart (Figure 1). A brief description of the main features of the 26 articles found is provided in Table 3 (Table 3).

All the articles included were assessed according to the PEDro scale which assigned a score of 0 if the criterion is absent and 1 if it is present in the article [9]. The first criterion on this scale, reporting eligibility criteria, was not recorded because it considered the external validity of the articles. The conduct and design of study are evaluated by eight items (item 2–9). Item 10 involves reporting between-group statistical comparisons, and item 11 involves measures of variability [10]. On this scale, studies with scores higher than 9 points are considered to have excellent methodological quality, those with scores between 6 and 8 points are deemed good, those from 4 to 5 points are considered to have regular quality and those below 4 are regarded as having poor methodological quality. 

**Table 3:** Description of articles included in the systematic review.

Author	Year	Type	Sample	Objectives	Results	Conclusions
Automatic detection of diabetic foot complications with infrared thermography by asymmetric analysis						
Liu, C.; van Netten, J.J.; van Baal, J.G.; Bus, S.A.; van der Heijden, F. [3]	2015	Asymmetric analysis.	76 patients with DM and diabetic foot complications.	Perform simple asymmetric analysis between the left and right foot combined with foot segmentation based on color images and non-rigid registration according to landmarks.	A segmentation of the feet was performed and infrared and color images were obtained.	Comparison of color and thermal images allowed the identification of common points between both materials.
2. Infrared thermography and vascular disorders in diabetic feet						
Ilo, A.; Romsi, P.; Mäkelä, J. [5]	2020	Case-controlled study.	118 patients with DM and 93 healthy individuals.	Evaluate the diagnostic potential of a novel non-invasive diagnostic method, IRT, compared with the conventional non-invasive method (ankle-brachial index and pressure on the 1st finger) in 5 study areas.	Patients with DM generally had warmer feet with a significantly higher temperature. IRT revealed differences between angiosomal areas, subclinical infections, and high-pressure plantar areas.	IRT revealed local temperature differences in high-risk diabetic feet. However, it is important to combine its use with other traditional screening methods.
3. A medical thermal imaging device for the prevention of diabetic foot ulceration						
Machin, G.; Whittam, A.; Ainarkar, S.; Allen, J.; Bevans, J.; Edmonds, M.; Kluwe, B.M.A.; Petrova, N.; Plassmann, P.; Ring, F.; et al. [11]	2019	Descriptive, observational study.	103 healthy volunteers (50 men and 53 women).	Describe the development, characterization, and initial results of a thermal imaging device aimed at significantly reducing the incidence of DRFUs.	Healthy feet are thermally symmetrical. In some participants, there were differences of >2.2 °C between the same site on both feet. After 10 min, these differences had significantly reduced.	These thermal imaging devices were shown to be fit for purpose and could identify areas of concern in the foot. This device could also be beneficial in other clinical settings such as in the study and prevention of pressure ulcers.
4. Reliability of a novel thermal imaging system for temperature assessment of healthy feet						
Petrova, N.L.; Whittam, A.; MacDonald, A.; Ainarkar, S.; Donaldson, A.N.; Bevans, J.; Allen, J.; Plassmann, P.; Kluwe, B.; Ring, F.; et al. [12]	2018	Multicenter clinical trial.	52 men and 53 women aged 18 to 69 years.	Explore the reliability of this device for assessing the temperature of healthy feet.	There was substantial-to-perfect inter-instrument agreement between the handheld thermometer and thermal imaging device, with the intra-class correlation coefficients in the 5 regions of interest ranging from 0.94 to 0.97.	The thermal imaging device showed exceptionally good agreement over repeated evaluations. Additionally, it could provide an instantaneous thermal image of all the sites on the feet.
5. Reproducibility of thermal images: some healthy examples						
Macdonald, A.; Petrova, N.; Ainarkar, S.; Allen, J.; Plassmann, P.; Whittam, A.; Bevans, J.; Ring, F.; Kluwe, B.; Simpson, R.; et al. [13]	2017	Comparative, observational study.	30 healthy participants.	Investigate the use of thermal imaging in the treatment of patients at a high risk of developing DRFUs.	The feet were thermally symmetrical, although the absolute temperature varied between visits. Temperature differences at specific locations on the foot exceeded the threshold of 2.2 °C.	These studies provide a basic understanding of thermal symmetry in the feet of healthy participants that can be used when interpreting images of the feet of patients with DM and DPN.
6. Validation of low-cost smartphone-based thermal camera for diabetic foot assessment						
Van Doremalen, R.F.M.; van Netten, J.J.; van Baal, J.G.; Vollenbroek-Hutten, M.M.R.; van der Heijden, F. [14]	2019	Simple study.	32 participants.	Validate a smartphone-based high-end infrared camera for the assessment of diabetic foot.	Near-perfect agreement for the temperature measurements, both throughout the plantar foot and in pre-specified regions.	The validity of the smartphone-based infrared camera was excellent for assessing diabetic foot.
7. Infrared thermography and ulcer prevention in the high-risk diabetic foot: data from a single-blind multicentre controlled clinical trial						
Petrova, N.L.; Donaldson, N.K.; Tang, W.; MacDonald, A.; Allen, J.; Lomas, C.; Leech, N.; Ainarkar, S.; Bevans, J.; Plassmann, P. [15]	2020	Single-blinded, multicenter clinical trial.	110 patients with diabetes mellitus (DM), diabetic peripheral neuropathy (DPN), and a history of diabetes-related foot ulcers (DRFUs).	Evaluate the usefulness of thermography and standard foot care in reducing DRFU recurrence.	After 12 months, 62% of the participants in the intervention group and 56% in the control group were ulcer-free.	Monthly intervention with thermal imaging did not result in a significant reduction in the ulcer recurrence rate or increased ulcer-free survival. However, a refined study with a longer follow-up and group stratification was planned.
8. Infrared thermal imaging for automated detection of diabetic foot complications						
Van Netten, J.J.; Van Baal, J.G.; Liu, C.; Van Der Heijden, F.; Bus, S.A. [16]	2013	Pilot study.	15 diabetic patients.	Explore the applicability of infrared thermal imaging for non-invasive automated systems.	Differences in the average temperature between the ipsilateral and contralateral foot were a maximum of 1.5 °C. The difference in patients with complications was at least 3 °C, with the feet of patients with Charcot–Marie–Tooth disease or osteomyelitis being warmer, and those with critical ischemia being colder compared to the contralateral foot.	An algorithm that could detect signs of diabetic foot disease and discriminate between non-local or diffuse diabetic foot complications was found. This algorithm was based solely on parameters that can be captured and analyzed with an infrared camera and a computer.
9. Infrared 3D thermography for inflammation detection in diabetic foot disease: a proof of concept						
Van Doremalen, R.F.M.; van Netten, J.J.; van Baal, J.G.; Vollenbroek-Hutten, M.M.R.; van der Heijden, F. [17]	2020	Single-center, prospective, cross-sectional study.	8 diabetic patients with a DRFU.	Explore the importance of 3D viewing of thermal imaging models for the detection of inflammation in diabetic foot disease.	Color definition maps were combined with thermal infrared images to create the first 3D infrared thermography (IRT) images of diabetic feet. Validity was evaluated +− 6 and +− in 2 cases.	3D viewing of thermographic images was clinically useful for the detection of inflammation.
10. Is thermal imaging a useful predictor of the healing status of diabetes-related foot ulcers? A pilot study						
Aliahmad, B.; Tint, A.N.; Arjunan, S.P.; Rani, P.; Kumar, D.K.; Miller, J.; Zajac, J.D.; Wang, G.; Ekinci, E.I. [18]	2018	Prospective, observational study.	Thermal and color images of 26 neuropathic DRFUs in people with type-1 or 2 DM.	Predict the healing of DRFUs using thermal imaging within the first 4 weeks of ulceration.	For the cases that healed, the ratio of wound bed area to baseline wound area measured by thermal imaging was significantly lower at 2 weeks compared to cases that did not heal.	This demonstrated that the change in the isothermal area of DRFUs can predict their healing status. DRFU thermal imaging has the advantage of incorporating both area and temperature, allowing the early prediction of healing of these ulcers.
11. Between visit variability of thermal imaging of feet in people attending podiatric clinics with DPN at high risk of developing foot ulcers						
Macdonald, A.; Petrova, N.; Ainarker, S.; Allen John Lomas, C.; Tang, W.; Plassmann, P.; Ehittam, A.; Bevans, J.; Ring, F.; Kluwe, B. [19]	2019	Observational, prospective study.	96 patients DRFUs.	Quantify the inter- and intra-patient thermal variations presented in diabetic feet with a high risk of ulceration.	The variation in right/left temperature differences for patients between visits was comparable to the variation observed between patients.	Thresholds that depend on thermal differences from one visit to another are unlikely to be sufficiently specific to effectively target treatments designed to prevent the development of DRFUs.
12. The application of medical thermography to discriminate neuroischemic toe ulceration in the diabetic foot						
Gatt, A.; Falzon, O.; Cassar, K.; Camilleri, K.P.; Gauci, J.; Ellul, C.; Mizzi, S.; Mizzi, A.; Papanas, N.; Sturgeon, C.; et al. [20]	2018	Prospective study.	12 patients with type-2 DM recruited from a hospital.	Determine if thermography can detect temperature differences between healthy feet, non-ulcerated neuroischemic feet, and neuroischemic feet with toe ulcers in patients with type-2 DM.	There was a significant difference in toe temperature between these 3 groups.	First study to examine the thermographic patterns related to the toe temperature of patients with neuroischemic ulceration compared to non-ulcerated neuroischemic and healthy feet in patients with type-2 DM.
13. Comparison of thermal foot maps between diabetic patients with neuropathic, vascular, neurovascular, and no complications						
Astasio-Picado, Á.; Martínez, E.E.; Gómez-Martín, B. [21]	2019	Descriptive, cross-sectional, observational study.	277 patients with a diabetic pathology.	Use IRT to analyze temperature differences between the feet of users with DM with DPN, vasculopathy, neurovascular disease, or none of the above, by segmenting the sole of the foot into 4 areas for the purposes of the study.	Lower temperatures under the ball of the big and little toes, heel, and pulp of the big toe in patients with DM compared to the healthy group.	IRT may be useful in evaluating the foot at risk in order to reveal temperature variability depending on the area under study.
14. Thermal map of the diabetic foot using infrared thermography						
Astasio-Picado, A.; Martinez, E.E.; Nova, A.M.; Rodriguez, R.S.; Gomez-Martin, B. [22]	2018	Descriptive, cross-sectional, observational study.	277 diabetic patients.	Study the use of IRT in the analysis of foot temperature variability in diabetic patients by segmenting the sole of the foot into 4 areas of interest.	The technique distinguishes any temperature variability between the different study areas of the soles of each foot.	IRT can provide useful clinical information to aid in the early diagnosis and prevention of lesions to compromised areas of the foot.
15. Morphological pattern classification system for plantar thermography of patients with diabetes						
Mori, T.; Nagase, T.; Takehara, K.; Oe, M.; Ohashi, Y.; Amemiya, A.; Noguchi, H.; Ueki, K.; Kadowaki, T.; Sanada, H. [23]	2013	Cross-sectional, observational study.	32 healthy individuals and 129 patients with DM.	Evaluate individual thermographic variations and compare them with angiosome results obtained in previous studies.	Different thermographic patterns (whole pattern and butterfly pattern) were compared between diabetic and non-diabetic patients.	The system, which was based on IRT, was useful for screening the circulatory status in patients with DM.
16. Early diagnosis of DPN based on infrared thermal imaging technology						
Zhou, Q.; Qian, Z.; Wu, J.; Liu, J.; Ren, L.; Ren, L. [24]	2021	Case-controlled study.	60 patients with mild DPN and 60 healthy volunteers.	Detect and compare the surface temperature of the plantar vessels in patients with mild DPN and healthy controls.	Excellent test–retest reliability, with differences in skin temperature between patients with mild NPD and healthy controls.	They provided a convenient, non-invasive, in vivo approach and methods for the early diagnosis of DPN.
17. Plantar temperature and vibration perception in patients with diabetes: a cross-sectional study						
Bhargavi, A.; Anantha, K.; Janarthan, K. [25]	2020	Case-controlled study.	50 healthy individuals and 50 patients with DM.	Correlate the temperature and vibration sensitivity values obtained from the same patient to reduce false positive results in the diagnosis of DRFUs.	The perception of vibration was compared with the points with the highest temperature on the contralateral feet.	More variables need to be compared to obtain better results for the classification of lesions in pre-ulcerative stages.
18. Early detection of foot ulceration in type II diabetic patient using registration method in infrared images and descriptive comparison with deep learning methods						
Rai, M.; Maity, T.; Sharma, R.; Yadav, R.K. [26]	2022	Observational, comparative study.	60 people (37 men and 23 women).	Early diagnosis and minimization of the appearance of DRFUs with the use of IRT.	The results clearly distinguished the foot region which showed a temperature difference higher than the assumed threshold value.	This analysis clearly classified the foot as at risk of ulceration and it was quite easy to understand compared to existing deep learning techniques.
19. Morphological foot model for temperature pattern analysis proposed for diabetic foot disorders						
Arteaga-Marrero, N.; Bodson, L.C.; Hernandez, A.; Villa, E.; Ruiz-Alzola, J. [27]	2021	Comparative, observational study.	9 healthy women and 13 healthy men.	Provide a methodology to explore all the plantar aspects of both feet, based on IRT, for the evaluation of diabetic foot anomalies.	Comparison of both feet to register patterns; differences were observed between men and women.	A quick and easy monitoring tool was provided for diagnostic use in patients with diabetic foot disorders.
20. Plantar thermogram database for the study of diabetic foot complications						
Hernandez-Contreras, D.A.; Peregrina-Barreto, H.; Rangel-Magdaleno, J.D.; Renero-Carrillo, F.J. [28]	2019	Descriptive, observational study.	334 plantar thermograms.	Use thermograms to collect images corresponding to the 4 plantar angiosomes in order to create a database.	Describes the plantar thermogram acquisition protocol, including the acquisition system and proper preparation of patients.	It was hoped that the database would become a valuable resource to promote research into the potential of IRT for the early diagnosis of diabetic foot problems.
21. Thermographic characteristics of the diabetic foot with peripheral arterial disease using the angiosome concept						
Carabott, M.; Formosa, C.; Mizzi, A.; Papanas, N.; Gatt, A. [29]	2021	Comparative study.	27 participants.	Compare temperature changes in 3 forefoot angiosomes after a limb elevation challenge between type-2 DM patients with and without peripheral arterial disease (PAD).	The mean resting temperature for all the angiosomes of participants with PAD were higher than for those without PAD. There was a significant difference in the initial mean temperature between the groups in the medial and lateral forefoot angiosomes.	Patients with PAD exhibited significantly higher forefoot temperatures, according to the analysis applying the angiosome concept.
22. Use of smartphone attached mobile thermography assessing subclinical inflammation: a pilot study						
Kanazawa, T.; Nakagami, G.; Goto, T.; Noguchi, H.; Oe, M.; Miyagaki, T.; Hayashi, A.; Sasaki, S.; Sanada, H. [30]	2016	Pilot study.	16 images.	Verify the reliability and validity of the FLIR ONE^®^ camera for evaluating inflammation based on the relative temperature increase compared to thermography when routinely used in the evaluation of pressure ulcers and diabetic foot.	An analysis of 16 thermal images.	This study suggested that FLIR ONE^®^ may function as an alternative device for the evaluation of subclinical inflammation in pressure ulcers and diabetic foot in clinical settings.
23. Prevention of diabetic foot ulcers using a smartphone and mobile thermography: a case study						
Oe, M.; Tsuruoka, K.; Ohashi, Y.; Takehara, K.; Noguchi, H.; Mori, T.; Yamauchi, T.; Sanada, H. [31]	2021	Case study.	2 patients.	Evaluate the effects of a self-monitoring device to prevent diabetic foot ulcers using a thermographic camera connected to a smartphone.	One patient is able to detect an increase in temperature when walking while the other one cannot.	This device can be used for self-care, although it is important to modify it for use in high-risk situations.
24. Evaluation of the surface temperature distribution in the feet of patients with type 2 diabetes using the thermal imaging method						
Dębiec-Bąk, A.; Skrzek, A.; Ptak, A.; Majerski, K.; Uiberlayová, I.; Stefańska, M. [32]	2023	Case-control.	52 diabetic subjects and 33 controls.	Superficial dorsal and plantar temperature of both feet was measured in all participants using a thermal imaging device.	On average, diabetic patients dorsal and plantar temperature were 2.2 °C and 1.5 °C higher, respectively.	Thermography in diabetic patients can be a useful tool to detect neurotrophic changes in the feet.
25. Mobile Application for Ulcer Detection						
Fraiwan, L.; Ninan, J.; Al-Khodari, M. [33]	2018	Cross-sectional study.	4 images were analyzed: one healthy foot and the other 3 were ulcer-simulated situations	Develop a mobile application to detect potential ulcers using a smartphone connected to a thermal camera. A mean temperature difference greater than 2.2 °C was considered as an indicator of possible ulcer development.	The app detected one image with a mean temperature difference lower than 2.2 °C and the other three greater, as expected.	Temperature monitoring of the feet in diabetic patients can help in the diabetic foot ulcer prevention. This app is a promising tool.
26. Monitoring of pH and temperature of neuropathic diabetic and nondiabetic foot ulcers for 12 weeks: An observational study						
Gethin, G.; O’Connor, G.M.; Abedin, J.; Newell, J.; Flynn, L.; Watterson, D.; O’Loughlin, A. [34]	2018	Case-control.	50 neuropathic patients: (34 with diabetes).	Determine baseline date regarding ph, size and temperature of non-infected neuropathic foot ulcer surfaces. Survey changes for 12 weeks looking for the changes in these characteristics related to the healing of the ulcer.	62.5% of the cases where the ulcer was healed had a reduction in the central temperature of the wound.	Reference values of the thermal variation are given and can be used in greater cohort studies.

In this review, only one paper was considered to have excellent methodological quality, and three studies were rated as having a good quality. The majority fell within the range of 5–4 points. Only four references scored below 4 points, indicating poor methodological quality (Table 4). 

## 4. Discussion

To assess if infrared thermography was a useful tool for diagnosing pre-ulcerous states and detecting the complications of ulcers in diabetic patients, we performed an exhaustive review of the relevant available literature. We found elements that made this comparison difficult, such as the differences in the samples and methodology used in each study, including the infrared camera employed and the protocol followed to capture the images. In spite of these challenges, we have emphasized the lack of evidence for the use of thermography in preventing complications, the absence of consensus in the use of angiosomes and temperature distribution patterns, as well as the need to establish thermographic standards that facilitate the comparison of obtained data.

Our search highlighted articles that had analyzed and compared foot temperature using infrared thermography in healthy [11,12,13,14] and diabetic patients [3,5,14,15,16,17,18,19,20,21,22,23], although other studies had directly analyzed both types of patients [24,25,26,27,28,29,30,31,32,33,34]. This type of comparison had confirmed the difference between the temperature of both groups, registering lower temperatures in the healthy group as compared with the diabetic one [3,5,24,25,26,27,32,33]. However, it is important to consider the results obtained depend on the protocol used. Indeed, studies such as those by Machin et al. [11] and Rai et al. [26] observed an increase in plantar temperature in healthy patients that could be indicative of the presence of inflammation. The previous publication of benchmark values in healthy people [11,12,13] allowed us to define standardized results that could be used to refine the methodology to achieve satisfactory results when taking samples. 

Studies in diabetic patients have highlighted the fact that thermography can be used as a method of temperature control (which is approximately 2 °C higher in infected wounds) useful to help patients in avoiding pressure zones, thereby favoring the prevention of lesions and their complications [16,34]. However, other authors such as Petrova et al. [15] found no difference in ulcer appearance in diabetic patients allocated in a randomized control trial where one group was blinded to the results of the thermography and the other was not (both receiving also standard care and follow-up). Therefore, there is insufficient evidence to support the use of thermography to prevent complications because the values it detects do not always significantly differ from normality and studies with larger sample sizes are still required. Databases such as the one published by Hernández-Contreras et al. [28] which contain both healthy and diabetic patient thermograms could be created. This database contains 167 patients and so could serve as a useful baseline for the comparison of results with those of future studies. Although different types of voluntary subjects were included in the different studies considered in this review, these results could be addressed together because they all suggest that thermography is a suitable tool to detect foot temperature changes. If they are detected sufficiently early, this technology may help to prevent the appearance of pre-ulcerous complications and could favor ulcer healing and avoiding infections through early treatment [15,18,25,34].

Even though one of the exclusion criteria for this current work was not being exposed to other pathologies that could result in temperature changes, it was very difficult to isolate diabetic foot from other pathologies such as peripheral vascular disease because many of these coexist [25,28,29,31]. Indeed, some authors observed that temperatures in the fifth metatarsal and hallux were lower in patients with associated pathologies [5,21]. 

Furthermore, Mori et al. [23] suggest that temperature distribution patterns must be identified by collecting and classifying these images according to maps of heat distribution so that they can be compared without relying on the angiosome divisions proposed by other authors [5,23]. Other authors suggest that patients with diabetic foot present a significantly higher temperature in the fifth metatarsal area and hallux compared to healthy individuals [20,22,27]. These variations represent different pattern distributions of temperature. For example, diabetic patients have notably higher temperature asymmetry and hot spots on the sole of the foot that indicate a heat increase [11,26]. Regardless, the lack of consensus in the use of angiosomes and temperature distribution patterns make it difficult to generalize the results published up to date. Moreover, it is important to highlight the differences in thermographic images from studies comparing the affected and contralateral limbs in diseased or healthy patients. According to Van Netten et al. [16], there was no difference between the limbs of the same patient, while Macdonald et al. [20] or Mori et al. [23] suggested that intra-patient variability is comparable with that of other patients, with a difference of up to 2 °C between both feet. Nonetheless, infrared thermography is the most common method used to compare contralateral limbs in these patients [13,15,16,19,22]. The work by Gatt et al. [20] indicated that there were differences between patients with or without lesions. This increased temperature may be altered in pre-ulcerous states, although once the wound is present it is difficult to predict the development of complications using thermography alone, because no difference between ulcered and healthy toes of the same foot can be detected. However, Gethin [34] is able to show a progressive core temperature reduction along with the healing of the wound.

As previously mentioned, not only the study population is variable in the studies reported, but also the characteristics of the thermal imaging device and methods of image acquisition. A wide range of different infrared cameras are used for these types of study. On average, it can be assumed that a standard spatial resolution of 320 × 240 pixels [15,20,22,26] conferees a thermal resolution of up to 0.1 °C [16,19,20,25] with a temperature range of 0–100 °C [30]. Although the use of standard infrared cameras is quite common, some studies have proposed using infrared smartphone cameras [14,30,31,33] to make this technique even more accessible. Indeed, some studies have even proposed merging 3D and thermography images to obtain more realistic results [16], as well as developing mobile apps able to detect temperature changes. [31,33]. However, phone cameras have a lower (160 × 120 pixels) resolution [14,17] and these devices cannot obtain specific data like absolute temperature. Despite this limitation, Kanazawa et al. [30] suggest that images are obtained much faster and are sufficient for data comparison. In fact, Van Doremalen et al. [14] stated that cameras connected to smartphones showed excellent validity for studying diabetic foot and therefore, such studies with smartphone cameras should be increased. Of note, although working at room temperature, some studies use a black background to dampen the influence of light [3,14,22,25,26] because environmental variations can alter the results, as shown by Bhargavi et al. [25]. 

As it has been observed during the development of this current study, there are some limitations to comparing the use of infrared thermography in patients with diabetic foot. First, very few studies have individually tackled this technique, comparing it with other techniques or in other pathologies associated with diabetic foot. Second, a wide range of cameras can be used to record sample images, leading to the misconception that accuracy is unreliable, but actually, images are not necessarily comparable, depending on many factors, especially their spatial resolution. Furthermore, the decision to use a black background in the images can significantly alter the results and no algorithms have yet been developed that can unify all these criteria. 

In addition, it is to be highlighted that working with healthy or diabetic individuals with or without an ulcer or underlying pathologies can result in very disparate sample images. Thus, it has been proposed by other authors, the generation of a large thermographic image database could help to create thermographic standards and to make the comparison of results more feasible. Homogenous criteria that can help to unify results must be developed so that this technique can be used to help prevent complications or promote ulcer healing in diabetic patients.

## 5. Conclusions

Infrared thermography represents a potentially effective tool for detecting pre-ulcerous states in diabetic foot patients. However, further research is still required to investigate its applications for preventing diabetic foot ulcer complications. It is very important to highlight that these thermographic studies must consider the sample type (healthy or diabetic patients, with or without an ulcer), protocol used to obtain the images (e.g., with or without a black background), and results analysis (software used). It is essential to continue research into infrared thermography, especially with the aim of clarifying whether the use of angiosomes or patterns of plantar temperature distribution is better; the influence of peripheral vascular disease on variations in plantar temperature in the diabetic population; and the reliability of cameras connected to smartphones. Infrared thermography is a cheap, non-invasive, and painless technique that can provide useful data for the development of new methods to further prevent and palliate diabetic foot complications.

## Figures and Tables

**Table 1:** PICO research questions.

P	Patient	Healthy or diabetic patients with or without ulcers.
I	Intervention	Diagnosis of diabetic foot complications.
C	Comparison	Use of thermography as a diagnostic tool for complications versus not using this technology.
O	Outcomes	Early detection of complications.

**Table 2:** Bibliographical database search strategy.

Database	Search Strategies	Data	Results
PubMed	((((“Diabetic Foot”[Title/Abstract]) OR (diabetic foot[MeSH Terms])) OR ((“Diabetic Neuropathies”[Title/Abstract]) OR (diabetic neuropathies[MeSH Terms])))) AND (((“thermal imaging”[Title/Abstract]) OR (differential thermal analysis[MeSH Terms])) OR (analyses, differential thermal[MeSH Terms]))	September 2023	30
WoS	(“Diabetic Foot” OR “Diabetic Neuropathies”) AND (“thermal imaging” OR “differential thermal analysis”)	September 2023	61
Cochrane	((diabetic foot) OR (neuropathy)) and ((thermal imaging) OR (infrared thermography) OR (temperature monitoring) or (infrared image) OR (skin temperature) OR (thermal imaging) OR (infrared sensor technology))	September 2023	224
SCOPUS	(((diabetic AND foot) OR (neuropathy)) AND ((“thermal imaging”) OR (“infrared thermography”) OR (“temperature monitoring”) OR (“infrared image”) OR (“skin temperature”) OR (“thermal imaging”) OR (“infrared sensor technology”)))	September 2023	884

**Table 4:** Articles’ methodology quality scores according to the PEDro scale. “✓” Meet requeriment and “✗” do not meet requeriment.

	Inclusion and Source	Random Assign	Hidden Assign	Baseline Comparability	Blinded Subjects	Blinded Therapists	Blinded Raters	Results above 85%	Analysis by “Intention to Treat”	Statistical Comparisons between Groups	Measurement and Variability Data	SCORE
Liu, C.; van Netten, J.J.; van Baal, J.G.; Bus, S.A.; van der Heijden, F. (2015) [3]	✓	✗	✗	✗	✗	✗	✗	✓	✓	✓	✓	4
Ilo, A.; Romsi, P.; Mäkelä, J. (2020) [5]	✓	✗	✗	✓	✗	✗	✗	✓	✓	✓	✓	5
Machin, G.; Whittam, A.; Ainarkar, S.; Allen, J.; Bevans, J.; Edmonds, M.; Kluwe, B.M.A.; Petrova, N.; Plassmann, P.; Ring, F.; et al. (2019) [11]	✓	✗	✗	✓	✗	✗	✗	✓	✓	✓	✓	5
Petrova, N.L.; Whittam, A.; MacDonald, A.; Ainarkar, S.; Donaldson, A.N.; Bevans, J.; Allen, J.; Plassmann, P.; Kluwe, B.; Ring, F.; et al.(2018) [12]	✓	✗	✗	✓	✗	✗	✗	✓	✓	✓	✓	5
Macdonald, A.; Petrova, N.; Ainarkar, S.; Allen, J.; Plassmann, P.; Whittam, A.; Bevans, J.; Ring, F.; Kluwe, B.; Simpson, R.; et al. (2017) [13]	✓	✗	✗	✓	✗	✗	✗	✓	✓	✓	✓	5
Van Doremalen, R.F.M.; van Netten, J.J.; van Baal, J.G.; Vollenbroek-Hutten, M.M.R.; van der Heijden, F. (2019) [14]	✓	✗	✗	✓	✗	✗	✗	✓	✓	✓	✓	5
Petrova, N.L.; Donaldson, N.K.; Tang, W.; MacDonald, A.; Allen, J.; Lomas, C.; Leech, N.; Ainarkar, S.; Bevans, J.; Plassmann, P. (2020) [15]	✓	✓	✓	✓	✓	✗	✗	✓	✓	✓	✓	8
Van Netten, J.J.; Van Baal, J.G.; Liu, C.; Van Der Heijden, F.; Bus, S.A. (2013) [16]	✓	✗	✓	✓	✗	✗	✗	✓	✓	✓	✓	6
Van Doremalen, R.F.M.; van Netten, J.J.; van Baal, J.G.; Vollenbroek-Hutten, M.M.R.; van der Heijden, F. (2020) [17]	✓	✗	✓	✗	✓	✗	✗	✗	✓	✓	✓	5
Aliahmad, B.; Tint, A.N.; Arjunan, S.P.; Rani, P.; Kumar, D.K.; Miller, J.; Zajac, J.D.; Wang, G.; Ekinci, E.I. (2018) [18]	✓	✓	✗	✓	✗	✓	✗	✓	✓	✓	✓	7
Macdonald, A.; Petrova, N.; Ainarker, S.; Allen John Lomas, C.; Tang, W.; Plassmann, P.; Ehittam, A.; Bevans, J.; Ring, F.; Kluwe, B (2019) [19]	✓	✓	✓	✓	✓	✗	✗	✓	✓	✓	✓	8
Gatt, A.; Falzon, O.; Cassar, K.; Camilleri, K.P.; Gauci, J.; Ellul, C.; Mizzi, S.; Mizzi, A.; Papanas, N.; Sturgeon, C.; et al. (2018) [20]	✓	✗	✗	✗	✗	✗	✗	✓	✓	✓	✓	4
Astasio-Picado, Á.; Martínez, E.E.; Gómez-Martín, B. (2019) [21]	✓	✗	✗	✓	✗	✗	✗	✗	✓	✓	✓	4
Astasio-Picado, A.; Martinez, E.E.; Nova, A.M.; Rodriguez, R.S.; Gomez-Martin, B. (2018) [22]	✓	✗	✗	✓	✗	✗	✗	✓	✓	✓	✓	5
Mori, T.; Nagase, T.; Takehara, K.; Oe, M.; Ohashi, Y.; Amemiya, A.; Noguchi, H.; Ueki, K.; Kadowaki, T.; Sanada, H. (2013) [23]	✓	✗	✗	✗	✗	✗	✗	✓	✓	✓	✓	4
Zhou, Q.; Qian, Z.; Wu, J.; Liu, J.; Ren, L.; Ren, L (2021) [24]	✓	✓	✓	✓	✓	✗	✗	✓	✓	✓	✓	8
Bhargavi, A.; Anantha, K.; Janarthan, K (2020) [25]	✓	✗	✗	✓	✓	✗	✗	✓	✓	✓	✓	6
Rai, M.; Maity, T.; Sharma, R.; Yadav, R.K. (2022) [26]	✓	✗	✗	✗	✗	✗	✗	✓	✓	✓	✓	4
Arteaga-Marrero, N.; Bodson, L.C.; Hernandez, A.; Villa, E.; Ruiz-Alzola, J. (2021) [27]	✓	✗	✗	✗	✗	✗	✗	✗	✓	✓	✓	3
Hernandez-Contreras, D.A.; Peregrina-Barreto, H.; Rangel-Magdaleno, J.D.; Renero-Carrillo, F.J (2019) [28]	✓	✗	✗	✗	✗	✗	✗	✗	✓	✓	✓	3
Carabott, M.; Formosa, C.; Mizzi, A.; Papanas, N.; Gatt, A. (2021) [29]	✓	✗	✗	✗	✗	✗	✗	✗	✓	✓	✓	3
Kanazawa, T.; Nakagami, G.; Goto, T.; Noguchi, H.; Oe, M.; Miyagaki, T.; Hayashi, A.; Sasaki, S.; Sanada, H. (2016) [30]	✓	✓	✓	✓	✓	✗	✓	✓	✓	✓	✓	9
Oe, M.; Tsuruoka, K.; Ohashi, Y.; Takehara, K.; Noguchi, H.; Mori, T.; Yamauchi, T.; Sanada, H. (2021) [31]	✓	✗	✗	✗	✗	✗	✗	✗	✓	✓	✓	3
Dębiec-Bąk, A.; Skrzek, A.; Ptak, A.; Majerski, K.; Uiberlayová, I.; Stefańska, M. (2023) [32]	✓	✗	✗	✓	✗	✗	✗	✓	✓	✓	✓	5
Fraiwan, L.; Ninan, J.; Al-Khodari, M. (2018) [33]	✓	✗	✗	✓	✗	✗	✗	✗	✓	✓	✓	4
Gethin, G.; O’Connor, G.M.; Abedin, J.; Newell, J.; Flynn, L.; Watterson, D.; O’Loughlin, A. (2018) [34]	✓	✗	✗	✓	✗	✗	✗	✓	✓	✓	✓	5

## Data Availability

Data are contained within the article.
